# Extracellular Vesicles-Based Drug Delivery Systems: A New Challenge and the Exemplum of Malignant Pleural Mesothelioma

**DOI:** 10.3390/ijms21155432

**Published:** 2020-07-30

**Authors:** Stefano Burgio, Leila Noori, Antonella Marino Gammazza, Claudia Campanella, Mariantonia Logozzi, Stefano Fais, Fabio Bucchieri, Francesco Cappello, Celeste Caruso Bavisotto

**Affiliations:** 1 Department of Biomedicine, Neuroscience and Advanced Diagnostics (BIND), Section of Human Anatomy, University of Palermo, 90127 Palermo, Italy; stefano.burgio94@gmail.com (S.B.); antonella.marinogammazza@unipa.it (A.M.G.); claudia.campanella@unipa.it (C.C.); fabio.bucchieri@unipa.it (F.B.); francesco.cappello@unipa.it (F.C.); 2 Department of Anatomy, School of Medicine, Tehran University of Medical Science, Tehran 141 765 3911, Iran; leili.noori.1359@gmail.com; 3 Department of Oncology and Molecular Medicine, National Institute of Health, 00161 Rome, Italy; mariantonia.logozzi@iss.it (M.L.); stefano.fais@iss.it (S.F.); 4 Euro-Mediterranean Institute of Science and Technology (IEMEST), 90139 Palermo, Italy

**Keywords:** extracellular vesicles, exosomes, drug delivery systems, malignant pleural mesothelioma

## Abstract

Research for the most selective drug delivery to tumors represents a fascinating key target in science. Alongside the artificial delivery systems identified in the last decades (e.g., liposomes), a family of natural extracellular vesicles (EVs) has gained increasing focus for their potential use in delivering anticancer compounds. EVs are released by all cell types to mediate cell-to-cell communication both at the paracrine and the systemic levels, suggesting a role for them as an ideal nano-delivery system. Malignant pleural mesothelioma (MPM) stands out among currently untreatable tumors, also due to the difficulties in achieving an early diagnosis. Thus, early diagnosis and treatment of MPM are both unmet clinical needs. This review looks at indirect and direct evidence that EVs may represent both a new tool for allowing an early diagnosis of MPM and a potential new delivery system for more efficient therapeutic strategies. Since MPM is a relatively rare malignant tumor and preclinical MPM models developed to date are very few and not reliable, this review will report data obtained in other tumor types, suggesting the potential use of EVs in mesothelioma patients as well.

## 1. Introduction

Cancer is a complex and multifactorial disease with both high incidence and mortality rates. Approximately 1 out of 6 deaths are caused by cancer, making it the second leading cause of death worldwide, with 9.6 million of deaths in 2018 [1]. Moreover, lung-associated cancers, with respectively 2.09 million worldwide cases and 1.76 million deaths in 2018 hold the primacy in terms of tumor-related morbidity and mortality [1]. In recent years, a new multidirectional approach has been adopted in oncology, by combining basic research aimed at enhancing our knowledge of the genetic and molecular characteristics that drive tumorigenesis and tumor growth with the attempt to develop new molecular targeting strategies for the treatment of pathology [2].

Over the years, therapies targeting tumor pathologies have become increasingly pioneering, in an attempt to overcome the limits imposed by classic chemotherapies, such as high toxicity, poor specificity and, consequently, a plethora of side effects. Such an approach, which is increasingly gaining ground, mainly concerns new delivery models and new therapeutic strategies that must be as specific and selective as possible, taking full advantage of the genetic/biomolecular differences of cancer cells compared to healthy tissues. Although survival rates have increased in recent years, and promising clinical trials for targeted drug delivery are ongoing, current treatments for many cancers remain ineffective and require the development of improved delivery methods. Indeed, in this review, we highlight the current “target-therapy” strategies, focusing our attention on the strengths and weaknesses of each approach. Moreover, we will focus on a particular tumor, the malignant pleural mesothelioma (MPM). MPM is a tumor with a poor prognosis. The surgical approach has significantly high perioperative mortality and high recurrence rate. In addition, chemotherapy is frequently only a palliative treatment due to the onset of chemoresistance, characteristic of this tumor. We therefore suggest the exploitation of extracellular vesicles (EVs) for the active delivery of therapeutic molecules. In fact, the intrinsic molecular characteristics of EVs, such as the high editability and low immunogenicity, make them the most suitable candidate. Currently there are no accessory therapies for MPM to be implemented if the patient develops resistance or conventional therapies do not prove effective. Therefore, since MPM has a “chameleon-like” extracellular profile that does not exhibit specific markers to be exploited for selective targeting, it is difficult to diagnose promptly. In this context, the use of a “Trojan horse”, self-produced by the tumor itself as a paracrine communication mechanism, is a real possibility. In fact, we reiterate that none of the current therapies or the ongoing clinical trials succeeds in what is the “unmet medical need”, that is, of therapeutic strategies aimed at prolonging the overall survival of the affected population, whose current average is estimated between 4 and 18 months [3].

The concept of “targeted therapy” was born due to the insights of Paul Ehrlich at the end of the 1800s, when he first theorized the use of a “magic bullet” [4,5]. Even though the idea of combining a cytotoxic molecule and a courier acting as a selective “bullet” was conceived for antimicrobial application purposes [4,5,6,7], over time it has increasingly become suitable in the cancer field.

The strategies adopted in recent years in order to accomplish the expectations on targeted therapy can be divided into two macro groups: on one hand, the first group involves the use of compounds or antibodies, which can interfere directly with the biology of the cancerous tissue or the tumor microenvironment; on the other, the second one concerns the developing of new drug delivery strategies, which can improve the biodistribution on the tumor site. However, together with the problems related to the best choice of a molecule to target in/on tumor cells, there are some issues that were and are unfortunately disregarded in thinking about future cancer therapies. The first and most important issue is related to the resistance of tumors to both chemical and biological treatments. Initially, this was thought to be due to a mechanism called multidrug resistance, through which a cancer cell is not responsive to most known drugs because of a group of proteins that are able to pump the drugs outside the cells; the most studied among these proteins was the P glycoprotein-1. This phenomenon of resistance of tumors to treatments has been described and characterized in several in vitro models. The experimentation in tumor patients is ongoing and it is aimed to improve the efficiency of anticancer drug delivery, overcoming the not responsiveness of tumor cells to conventional therapy [8]. In patients with some forms of leukemia, the drug resistance has shown to be dependent on the membrane-to-cytoskeleton connection through a family of proteins called ezrin/radixin/moesin [9]. This failure of scientific research seems to be due to the disregard of two important facts: (i) a chemical drug enters within a cell due to a pH gradient and (ii) all solid tumors live in an acidic microenvironment. In fact, being the vast majority of chemotherapeutics chemical drugs with an electric charge, and being virtually all weak bases, when they get to the acidic tumor microenvironment, they are protonated and therefore blocked outside the cells [8]. To support this finding, both buffers, such as sodium bicarbonate, and antiacidic drugs could highly improve the antiproliferative effect of antitumor drugs [8,10]. Particularly, proton pump inhibitors have been shown to significantly improve the effectiveness of antitumor drugs of different natures, including classical chemotherapeutics [11,12], drugs targeting surrogate tumor molecules [13] and small molecules as well [14]. Some clinical trials have supported the preclinical evidence [15,16,17], thus suggesting that this should represent at least one of the future anticancer strategies.

In this review, we provide the current state-of-the-art of strategies in cancer targeted therapy, with particular attention to EVs as new potential innovative nanocarriers with high targeting efficiency. We summarize the current knowledge on EVs’ biogenesis and features, believing in the importance of improving the understanding of their unique composition, in order to exploit them in clinical applications. Furthermore, we focus on the MPM whose management could be implemented by the application of EVs as a drug delivery strategy.

## 2. Strategies in Cancer Targeted Therapy

Patients’ response to treatments is related to the ability of the therapeutic system to be selective and to reach only the target tissue without affecting healthy sites. In order to improve response selectivity and to achieve a safer and more efficient systemic delivery, new technologies have been developed. These strategies, in particular in the oncological field, need to include a system able to release the active agent in a controlled manner and to target specific sites in the body, in order to realize an interface between the patient and the drug and then increase the effectiveness and reduce systemic effects.

Many new materials and approaches for drug delivery systems (DDS) are being developed. Among these, EVs appear to be promising candidates as nanocarriers over the conventional synthetic systems, considering their intrinsic features, including their tropism for specific organs or cells, their key role in intercellular communication and their non-toxicity. The concept of drug delivery is rooted in the early beginning of the 1950s. The first generation of DDS laid the groundwork of the controlled release of compounds, but it was from the second generation of systems (1980–2010) that researchers began to focus their attention on the setting up of smart DDS. Subsequently, from the third generation of DDS (from 2010), researchers tried to overcome both biological and physiochemical barriers, such as poor solubility of certain drugs, large molecular weight of protein-based drugs and the uncertain drug release in some kinds of formulations [18]. 

Therefore, in the last ten years precision medicine and nanotechnologies gave a strong contribution to the setting up of new delivery strategies, which may lead to a significant improvement of biodistribution and biocompatibility.

### 2.1. Nanoparticles

In the context of safe and effective therapeutic approaches, nanoparticles have received extensive interest as promising DDS for cancer treatment in recent years. Nanoparticles are the product of the applied research of nanotechnology, which is trying to cross both physical and biological barriers of the body in order to improve the drug delivery directly on the target site. In fact, when drugs are encapsulated in structures that are larger than five nanometers, this can easily prevent renal excretion, improving bioavailability [19].

Generally, nanoparticles can be defined as round nanospheres, built from synthesized nanomaterials, ranging between 100 and 1000 nanometers [20]. As they are nanosized, the uptake of nanostructures by cells is facilitated, allowing efficient drug delivery and ensuring target-specific action [21,22]. A plethora of materials have been used in nanosized carriers for cancer therapy, including polymers, lipids, protein–drug conjugates, viral nanoparticles, inorganic molecules and metal nanoparticles [23,24] (Table 1). Nanoparticles offer great advantages in nanomedicine applications, both in the diagnostic and therapeutic fields. In fact, the use of nano-systems is applied in early diagnosis, through non-invasive imaging modalities, for instance, by the use of fluorescent or magnetic systems. In the therapeutic field, research is trying to exploit their capability to trap, protect and deliver drugs to the target site, due to highly specific binding and internalization capabilities. Furthermore, it is possible to synthesize nanoparticles that have the possibility to integrate diagnostic and therapeutic entities within a single formulation, thus obtaining a single system capable of simultaneously satisfying the criteria of theranostic approaches and providing real-time feedback on pharmacokinetics, including the drugs’ target site localization and the off-target accumulation. These features are achieved due to both the co-loading of nanoparticles with drugs and with contrast agents, and the intrinsic ability of nanomaterials, such as gold and iron oxide-based nanoparticles, to be suitable to be used for the imaging [25,26].

However, it is important to underline that the use of some materials can be potentially risky for patients’ health [27]. In fact, carbon nanotubes, metal-based nanoparticles or even polymeric nanoparticles have been widely investigated and used for possible clinical applications. Despite the versatility of these therapeutic nanoscale agents used for the synthesis of nanomedicines, there are potential adverse side effects induced by the materials used [27]. In this regard, “green chemistry” approaches are increasingly considered in order to identify biocompatible/biodegradable nanomaterials capable of eliminating the toxicological impact and potential side effects deriving from some materials used, such as carbon nanotubes, metal nanoparticles or polymeric nanoparticles [27,28] (Table 1).

#### 2.1.1. Liposomes

Liposomes are lipid-based nanoparticles and were the nanoparticles used in nanomedicine [29]. They consist of a single or multiple lipid bilayer that is engineered to encapsulate, in the lipid bilayer or in the internal aqueous phase, hydrophobic or hydrophilic drugs or small molecules, depending on their polarity features [30]. Liposomes are the most investigated nanocarriers for targeted drug delivery, because of their morphological similarity with cellular membranes, allowing biocompatibility and minimal toxicity [31]. Furthermore, due to the possibility to modify their lipid bilayer characteristics and to build a large aqueous center, these nanoparticles permit to deliver a wide variety of macromolecules [32]. There are different classes of liposomes used as drug delivery systems, which are classified on the basis of size and number of layers; based on the composition, and on the method of preparation [33]. Moreover, in the last few years new liposome formulations have emerged, which are stimulus-sensitive and offer a more efficient drug release [34]. In a wide range of pathologies, liposomes seem to be effective as a target-therapy approach; indeed, it has been observed that they are able to improve and control pharmacokinetics and pharmacodynamics, to enhance drug activity, and are target selective. In cancer therapy, the use of liposomes offers several advantages, including efficient internalization by cancer cells that exploits the passive targeting effect, a phenomenon known as enhanced permeability and retention [35]. The in vitro and in vivo delivery efficacy of different liposomes formulations has been extensively studied in anticancer therapy and several data demonstrated low systemic toxicity and the capability to suppress tumor growth [36,37,38,39].

Despite the potential use of liposomes in the area of drug delivery, clinical applications have so far been negligible. In fact, there are some disadvantages limiting the development of liposome-based therapies, such as sensitivity to sterilization methods [40], stability issues [41], reproducibility in drug encapsulation, particle size control [42], short shelf-life and stability in blood circulation [43] (Table 1).

#### 2.1.2. Polymer-Conjugated Drugs for Selective Delivery

Ringsdorf properly described the concept of polymers and biopolymers-conjugated drugs in 1975 [44]. Water-soluble polymers are an efficient drug delivery system that can highly increase the half-life of compounds, significantly improve the general tolerance to high doses of drugs (such as chemotherapeutic drugs) and implement specificity. The first example reported in the literature of polymer-conjugated drugs is the dextran-methotrexate conjugate [45]. Kidney excretion of the chemotherapeutic drug was prevented, improving also the drug’s circulation in the blood stream. 

There are plenty of examples of peptides used as drug delivery vectors, which show promising results. Among these, the multiple antigen peptides (MAP) are one of them as they have both the specificity of an antibody and the versatility of a peptide. The branched structure of MAP peptides was originally designed to be used in the vaccination field [46,47]. However, in recent years the multimeric bond has been further analyzed in order to implement the avidity of the molecule. In fact, despite having a short amino acidic sequence and being in a branched form, it still was not able to trigger an effective immune response; the same cannot be said for the property of these molecules in terms of avidity and specificity. Therefore, if supported by a solid affinity and interaction study, target-peptide could become a reliable drug delivery model.

In this regard, an example could be the NT4, a tetra-branched form of neurotensin, which in the MAP form is correlated to a high affinity for the sulphate groups present in the glycosaminoglycans (GAGs) chains, highly expressed in most solid tumors [48,49,50].

It is possible to combine different chemotherapeutic drugs to the tetra-branched core of NT4, exponentially increasing their selectivity [48,51], as drug delivery to the target is strictly related to the carrier and with how much specificity it binds to its receptor (Table 1).

Progress achieved in recent years has made it possible to better implement and structure the NT4-based delivery system. In fact, the tetra branched peptide conjugated with the chemotherapy compound paclitaxel (PTX) was compared in an in vitro study with the PTX counterpart in the unconjugated form, demonstrating how the NT4-PTX complex can induce tumor regression, while the free PTX just led to a slowing down of tumor proliferation [51]. The delivery system was then refined and implemented, conjugating the NT4 peptide with NIR-emitting quantum dots nanoparticles (QD). The two tested formulations, NT4-QD and NT4-QD-PTX, have demonstrated effectiveness in vitro both in diagnosis/identification of the tumor itself and in theranostics on HT-29 cells, showing a higher cytotoxicity compared to the QD-PTX control-formulation used [52].

There are many possible applications of MAP peptides, including practical applications in the diagnosis of solid tumors [53]. However, practical applications are not limited to the oncological field: the properties of the multimeric bond are in fact provided by a huge variety of clinical contexts, such as in the implementation of the half-life of antibody fragments (Fab) injected intravitreally [54].

In this regard, the research group of Whitney Shatz et al., starting from a polyethylene glycol structure (PEG) backbone, synthetized an octameric Fab-PEG structure, capable of being administered intravitreally in order to treat age-related blindness pathologies. [54].

### 2.2. Small Molecules, Peptides and Antibody in Targeted Therapy

Small molecules are compounds whose molecular weight is generally <900 Daltons. Due to their small size, they can easily diffuse inside the cells, bind specific targets and expound their therapeutic function [55]. Small molecules in cancer carry out their function in different ways, such as intruding on the cell cycle, triggering apoptotic signals, and interfering with key enzymes, crucial for cell metabolism and slowing down tumor invasion and metastasis. Those molecules can also interfere with the tumor microenvironment, blocking the angiogenesis, boosting or regulating the immune system [56,57].

An analogue function is absolved by the monoclonal antibodies (mABs), largely used in clinics for a great variety of tumoral conditions. Those antibodies are developed starting from the hybridoma technology [58] and have been used in clinical trials since the 1980s [59]. MABs are usually produced for extracellular targets, and can fulfill their therapeutic function in both a direct and an indirect way. In the first case, the antibody is produced to recognize specific structures exposed on the tumor extracellular membrane, such as growth factor receptors or membrane bound proteins [60].

In the second case, mAB can be useful tools for the handling of tumor microenvironment, i.e., interrupting the interactions between ligands and their receptors (EGF, endothelial growth factor; vascular endothelial growth factor, VEGF; etc.), or promoting the recruitment of the immune system, in order to facilitate a direct and specific attack to cancerous cells.

Antibody-based therapy has deep roots in the history of biotechnologies. In fact, starting from serotherapy institutes (such as The Institute of Serotherapy and Vaccination of Tuscany), where active immunization against specific antigens was induced in large animals, such as horses, in order to use their plasma as a medical tool, subsequently we finally reached a more sophisticated biotechnological product [4,61].

The production of monoclonal antibodies originated in the early 1970s. Due to the two researchers Georges Kohler and Ceasar Milstein, the massive production of mABs was assessed using the hybridoma technology. The technology developed by the two researchers was never patented and it might be considered as a milestone in the large-scale production of mABs for therapeutic purposes. Therefore, due to the hybridoma technology, it was possible to produce and market the first murine mAB in 1986, “muromonab-CD3”, a full mouse mAB used to prevent kidney transplant rejection [62,63,64].

However, the clinical use of full murine antibodies has several limitations. The murine structure of the antibody might be recognized as “not self” from the patient, which may lead to a consistent immune reaction against the injected mAB.

Technological evolution and knowledge acquired in the field of genetic engineering have subsequently allowed one to create antibodies made of both murine genetic sequences and human genetic inserts, in a ratio of 33%–66%. Essentially these antibodies, which were defined as “chimeric”, presented a human Fc fragment, and a murine Fab. Antibodies thus constituted were less immunogenic than the murine counterpart was. The first chimeric antibody with these characteristics was approved for antithrombotic therapy in 1994, with the commercial name of Reopro^®^ (Abciximab) [4,64].

With the refinement of technology, it was possible to obtain chimeric antibodies containing less and less murine DNA, up to the so-called humanized antibodies consisting of 90–95% of human DNA sequences. In this typology of antibodies, the murine regions were located mostly in correspondence of the complementary-determining regions (CDRs). The first humanized antibody approved by the FDA in 1997, to prevent rejection following kidney transplants, was Zenapax^®^ (Daclizumab) [4,64].

Another important step forward in the production of mAB has been made through the introduction of the phage display technique. This technique allows the study of protein–protein interactions, starting from the genetic sequence of interest inserted within a viral vector, generally the bacteriophage M13. This protein of interest is exposed on the outer shape of the phage, preserving the genetic sequence of the interest inside. These phages are then subjected to interaction screening with the target protein, making possible large-scale affinity screening starting from genetic libraries, consisting of variants of the gene of interest [4,64,65].

Later in 2006, due to the phage display technology, it was possible to produce and market the first completely human monoclonal antibody, Humira^®^ (Adalimumab). The fully human antibody structure (100% compared to 90–95% of humanized ones) takes a step forward in the field of immunological therapy: murine CDR of humanized antibodies has been replaced by fully human CDRs, so that Adalimumab effectively improved compatibility and avoided structure-related immunogenicity.

The applications of mAB are various and can be used as powerful drug-carriers. Antibody-drug conjugates (ADC) are interesting tools in precision medicine, in which the mAB is covalently linked with a cytotoxic compound in order to be driven directly on the tumor site [60,66,67,68]. Of course, the above issue of acidity-mediated drug resistance still stands in the case of more targeted or smaller molecules, but for biological compounds as well, inasmuch as it appears highly conceivable that the acidic microenvironment may well hamper the affinity of a mAB for its specific epitope. Moreover, recent evidence has shown that microenvironmental pH markedly changes the lipid composition of tumor cells, thus hampering the hypothetical binding between a ligand and its receptor [69] (Table 1).

### 2.3. ADC

The therapeutic potential of antibodies has been deepened between the late 1800s and the early 1900s, when horses and oxen were exposed to an antigen in order to produce specific antibodies to it.

Despite the remarkable risk of rejection that the administration of animal immunoglobulins entailed, it is undeniable that such a practice has helped to encourage future studies and biotechnological innovations related to the engineering of antibodies for therapeutic purposes. Additionally, in the oncological field, the therapeutic applications of antibodies are several, as they can act as single entities, conjugated with a drug or an enzyme that in turn is capable of activating a drug, administered systemically [4]. There are several examples of antibodies that work as single entities, targeting a specific aberrant or overexpressed receptor component in neoplasms. An example may be Herceptin^®^, a humanized mAB for the treatment of metastatic breast cancer: it exerts its function binding the tyrosine-kinase receptor HER-2 present on the cell membrane.

Another example could be AVASTIN^®^ (Bevacizumab), a mAB aimed at the VEGF-A, proangiogenic growth factor. In combination with chemotherapy, it is approved for the treatment of advanced colorectal cancer, advanced non-small cell lung cancer and metastatic breast cancer [70,71,72].

Besides working as independent entities, several mABs exert their function conjugated with chemotherapeutic drugs or with radioactive ligands [73,74]. Indeed, numerous antibody-drug conjugates have been developed for the selective delivery of chemotherapeutic agents, and many of them have been tested in clinical trials; however, for some of them there was no significant improvement of the patient’s outcomes, compared to classical therapies [75,76,77,78] (Table 1).

### 2.4. Reconfigurable Organisms as Drug Carriers

A new promising study driven by the team of S. Kriegman et al. [79] opens up to a new way of conceiving nanomachines and, by extension, drug delivery. The research group focused its studies on a completely innovative model, which, starting from different biological tissues originating from *Xenopus laevis* embryos, gives rise to biological machines from scratch. Using compiler algorithms, 3D structures have been designed by the computer whose “blocks” are constituted by the epithelial and cardiac cells of the frog embryo. The most promising models are thus selected and used through the help of high precision manipulators, following the model path suggested by the computer. These “Xenobots”, thus defined by the research group, have several interesting peculiarities, including the ability to automatically self-repair, preserving their own integrity. The high motility is conferred by the cardiomyocytes content because the embedded cardiac cells make the structure self-propelled and establish a predictable path a priori. These structures, although still being in the early stages of research, have enormous potential, especially in the “drug delivery”, as they allow following a pre-established path and managing a load within the core [79] (Table 1).

## 3. EVs

EVs have recently entered solid tumor research, regarding the new possible targeted drug delivery systems, offering considerable advantages due to their intrinsic characteristics. Given this, and as the EVs are the object of our study, we devote a separate section for their discussion in this review.

EVs are lipid membrane vesicles actively secreted by all human cells and are involved in a plethora of cell-to-cell communication processes, both in pathological and physiological conditions. Although they were thought to be “garbage disposals” that eliminate unwanted cellular components, such as misfolded proteins or metabolic wastes [80,81], decades of research have defined their pivotal role in coagulation, cell-to-cell communication (both paracrine and systemic levels), vascular injuries and, more in general, in cellular maintenance of homeostasis [82].

EVs are actively secreted from all cell types, including cancer cells, and are highly heterogeneous in size, lipid layer composition and cargo (such as nucleic acids, proteins and lipids). In addition, EVs are classified according to their size: (1) microvesicles (100–1000 nm in diameter); (2) apoptotic blebs (1000–5000 nm in diameter) and (3) exosomes (diameter 20–150 nm). On one hand, the first two subclasses: they are a wide and heterogeneous population of vesicles originated from the outward budding of the cell membrane. On the other side, exosomes follow a different path, originating from the invagination of endosomal membranes. This invagination gives rise to multivesicular bodies defined by the presence of intraluminal vesicles (ILVs). The endosomal sorting complex required for transport (ESCRT) complex family mediates the inward folding of the membrane of the early endosomes, resulting in ILVs formation into the lumen [83,84,85,86]. The ESCRT is composed of four distinct proteins (ESCRT-0, -I, -II and -III), that accurately regulate biogenesis and cargo loading into the exosomes [87]. It is hypothesized that different structures for sorting molecules from cytoplasm toward exosomes may depend on their source cell. Additionally, the function of the cells can be understood via their released EV content [88].

Due to the heterogeneity existing within the EVs family, where boundaries between the various groups are often not easily distinguishable, a distinction is still problematic since the characteristics of these membranous vesicles overlap with each other, so in this review, we refer to EVs and exosomes commonly.

However, a specific type of EVs might be more represented rather than others, depending on the isolation method used. The EVs isolation methods development represents one of the greatest challenges in the case of the exploitation of EVs as therapeutic tools. Owing to the fact that the isolation protocol should achieve high efficiency, high purity and reproducibility. Among them, the gold standard in EVs isolation methods is represented by the differential ultracentrifugation, based on isolation by size. Over the years alternative methods have been developed, based on the size and the hydrodynamic radius, such as ultrafiltration, hydrostatic dialysis and gel filtration. Other approaches are precipitation methods, which exploit the variation of EVs solubility and/or their aggregation when placed in polymeric solutions, and lastly, methods utilizing affinity interactions, such as immunoaffinity techniques. Further information on technical issues on the isolation methods of EVs can be found in recent papers [89,90,91,92].

Each of these methods has its own advantages and weakness and the choice should be based on the sample type from which isolate EVs and depending on the final application. Nevertheless, due to the complexity of biological fluids from which EVs are being isolated, all the methods developed allow one to obtain heterogeneous mixtures of EVs and other extracellular space components. One solution may be the use of multiple isolation methods consecutively, in order to enrich a particular EVs population. Since the EV isolation efficiency is dependent on the nature of biological fluids, it is most important to standardize a particular method for isolation of EVs, taking into account specific characteristics of the sample, such as viscosity, typical of blood plasma and serum and, the presence of specific proteins, e.g., THP in the urine. Besides, the characterization of the obtained EV preparations, through the combination of different methods, such as electron microscopy, light scattering, flow cytometry and immunohistochemical analysis for the markers specific, is recommended to confirm the EV morphology, physical features and biochemical composition [83].

Exosomes are the main type studied among the family of EVs. They mediate the cell communication, and can be found in a large spectra of body fluids, such as blood, seminal fluid, urine, saliva, breast milk, ascitic fluid, bronchoalveolar lavage, malignant effusions, cerebrospinal fluid and amniotic fluid [88,93,94].

The intravesicular cargo may variate depending on the cell type that secretes them and according to their specific function on the recipient cell, such as cytokines [95], hormones [96], transcription factors, growth factors [97] and heat shock proteins [98,99,100,101,102,103,104,105,106,107]. Moreover, there is a large number of proteins that compose the structure of exosomes, which are directly involved in cell trafficking and their specificity: exosomes are enriched in proteins involved in the vesicles’ trafficking, cell surface receptors such as tumor susceptibility gene 101 (TSG101); integrin and a number of tetraspanins such as CD9, CD53, CD63, CD81 and CD82 [108,109]. Tetraspanin proteins are a functional ubiquitous region in the endosomal membrane, which assist in sorting of the cytosolic component into ILVs [110].

The release of EVs by tumor cells is believed to play a major role in intercellular communication, facilitating signaling to surrounding tumor cells and to distant sites via blood or other biological fluid transportation. Indeed, EVs cancerous cells can communicate with other tumor cells, with fibroblasts that surround the tumor, with endothelial cells and inflammatory cells, such as monocytes or T-cells [83].

As concerns fibroblasts, under tumorigenic conditions, they can be induced to change their morphological features, promoting a myofibroblast-like phenotype, more motile than the normal fibroblasts that surround the tumoral stroma. It has been proved that through the active secretion of EVs, tumoral cells can drive the polarization of normal fibroblasts into activated cancer-associated fibroblasts [111,112,113,114].

Strong evidence also underlines the EVs’ involvement in the angiogenesis in a plethora of tumor types [111,115,116,117,118]. The angiogenic activity is flanked by a pivotal role in the promotion of cell migration and metastasis in many tumor types [115], and the main mediators of this activity seem to be miR-9, miR-105, miR-142-3p, miR-210, miR-19a and H19 lncRNA [100,119,120,121,122,123].

For what may concern the immunomodulation, the literature has plenty of discordant results, which reflect the heterogeneity of EVs and their cell-specific function. Zhou M. and his research group show how pancreatic derived EVs mediated the expression of Toll-like receptor 4 (TLR4) in dendritic cells, promoting an antitumor immune response [86]. This strong evidence, taking in account the presence of major histocompatibility complexes on the surface of EVs that are also able to display cancer-derived peptides, suggested the possibility to develop an EV-based anticancer vaccine [124,125].

Despite those promising results, other evidence underlines how EVs have an inhibitory effect on immunomodulatory cells, especially on macrophages and dendritic cells [126,127].

Considering all the above, the possible therapeutic use of EVs in clinics becomes clear. 

Exosomes might be used as possible therapeutic targets in cancer, due to their confirmed role in tumorigenic progression, cancer cell communication and metastasis. In triple negative breast cancer (TNBC), for example, it has been proved that exosomes are involved in both the promotion of drug resistance and in the transferring of phenotypic traits from a progenitor cancer cell to another cell, thusly promoting cancer progression and metastasis [115]. The inhibition of exosome production in TNBC has been deepened through the blocking of ESCRT-independent pathways, or silencing the Rab27, which mediate another exosome-production pathway: in both cases, experimental evidence shows a slowing down of cell proliferation rate and reduction of local growth and metastasis [128,129].

Nevertheless, mostly and coherently with the aim of this review, exosomes might be a great potential drug delivery system [130,131]. Differently from what we have seen with the other abovementioned delivery systems, which in a few cases turned out to be poor in efficiency and high in immunogenicity and general tolerance [132], the endogenous origin of exosomes gives them a “boost” in terms of low immunogenicity and high specificity [115,133,134]. The intrinsic specificity of cancer-associated exosomes, which, as mentioned before, are the main characters in cancer cell-to-cell communication, make them powerful tools in the cancer-drug delivery system. Moreover, exosomes can be highly modified in order to enhance or modify their tissue-specificity, widening the amount of feasible therapeutic strategies [135]. Notably, preclinical studies have shown that exosomes are able to shuttle different kinds of molecules, including chemotherapeutics [136], drugs possibly useful to work as both effective molecules and tracers, representing a prototype for “Theranostics” [137,138], but nanoparticles as well [139], thus really supporting their exploitation as a natural delivery system for both diagnostics and therapeutics [140,141]. Moreover, their structure allows them to deliver active molecules as well, and this has been shown in in vitro models [142], and in vivo as well [143]. Lastly, it has been shown that exosome release and size are profoundly changed by extracellular acidity [144], suggesting that when using EVs or exosomes as a delivery system for the treatment of cancers the source of these should always be chosen very carefully [7] (Table 1).

### EV Bioengineering and Drug Loading

To consider developing EV-based therapy, it is necessary to take into account the cellular source from which to obtain them. Actually, although it is known that all cells can produce EVs, they should have some physical and biochemical characteristics that make them suitable as drug carriers. Among them, several features have been investigated, such as composition, influencing immunogenicity and targeting; loading capability, which affects therapeutic efficiency [145].

Currently, mesenchymal stem cell-derived EVs (MSC-EVs) are being studied and many experimental data have confirmed that these vesicles mimic the immune-regulating function and regenerative capacity of MSCs. The therapeutic potential of MSC-EVs, which exploit some intrinsic EV features, has been found in preclinical studies in various tissues, e.g., nervous tissue, cartilage and bone [146,147,148,149]. The mechanism of action of this type of EVs includes the activation of the immune system and the promotion of injury healing. In addition to the MSC-EVs, EVs from cells such as embryonic stem cells, induced pluripotent stem cells [150] and cardiomyocytes [151] have good therapeutic potential. In addition, dendritic cells have been used as EV cell sources in therapy, since they preserve the immunostimulatory functions of parental cells [152] and are able to cross biological barriers [153] with minor side effects [154]. Similar anticancer effects have been demonstrated for macrophage-derived EVs that modulate an immune response, including cell-mediated response against cancer cell growth [155,156]. Nevertheless, the role of macrophage-derived EVs appears ambiguous and it has been observed that they can favor both antitumoral and tumoral immune system stimulations [157].

As stated above, EVs, being carriers of natural active molecules including lipids, proteins, nucleic acids and other metabolites, have a broad therapeutic potential per se. However, for therapeutic applications, EVs should be obtained in significant amounts. Certainly, the achievement of clinically relevant doses highly influences EVs based therapy. Obtaining a sufficient quantity of EVs for clinical applications is influenced not only by the source, but also by the isolation processes and by the sample preservation and manipulation. Furthermore, these methods must ensure a high degree of quality, safety and consistency mandatory for clinical applications [158].

Although cell culture-derived EVs have been mainly used, other types of EVs can be easily isolated and manipulated, both in direct and indirect ways, to carry and deliver therapeutic molecules.

A possible approach consists of the isolation and engineering of cells from the patient, starting from body fluids [139,159] or from various foods including milk, fruits and vegetables [160,161]. Therapeutic compounds can be loaded within these EVs and infused into the patient.

The efficiency of this strategy is dependent on the kind of biological sample. Among the body fluids, blood plasma represents the most investigated EVs-source, both as a diagnostic tool (through a low invasive liquid biopsy) and as EVs producing cells reservoir. This because of the low invasiveness in obtaining it and because it contains cells that can be isolated and expanded in vitro for EVs production, and also because it contains a large amount of EVs produced by all the cells of our body and potentially shed into the bloodstream. For instance, monocyte-derived macrophages present in the blood have been shown to represent a safe and valuable source for therapeutic exosomes [162], and platelet-derived EVs that are rich in growth factors and noncoding RNAs [163,164]. These evidences support the idea that blood constitutes an almost ideal “EVs-source reservoir”, compared with other bodily fluids, for the production and isolation of clinical-grade EVs [158] both in terms of large-scale production and clinical feasibility [165].

**Figure 1:**
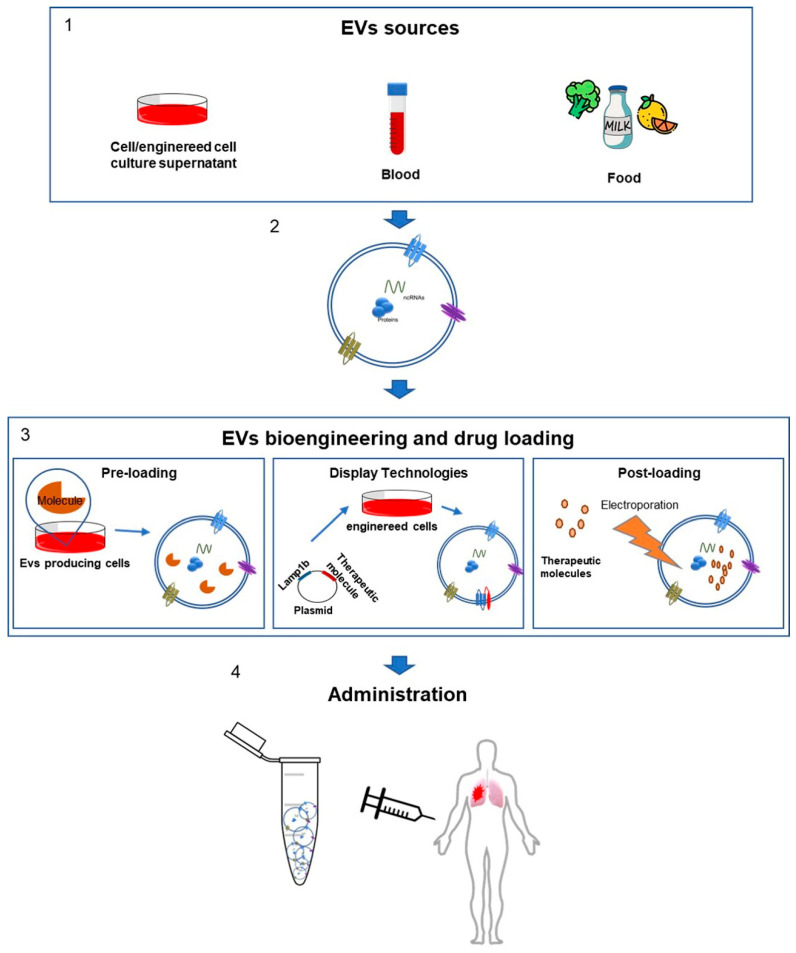
Strategy of EVs modification for therapeutic purposes. The figure schematizes the approaches currently used in EVs engineering or in the manipulation of their content. (**1**) The EVs are promising candidates in the treatment of numerous pathologies and there are various reliable sources. EVs can be isolated from the cell culture supernatant of various producing cell lines, from body fluids and also from food. (**2**) The EVs molecular composition is complex and it depends on the cellular source. They can contain different classes of proteins (membrane-bound tetraspanins CD9, CD81 and CD63; receptors, heat shock proteins), ncRNAs (RNAs; MicroRNAs) and lipids (lysobisphosphatidic acid; phosphatidylcholine; phosphatidylethanolamine and sphingomyelin). (**3**) The different methods to manipulate the EVs content include the preloading approach, in which a pre-existing endogenous cargo is the therapeutic molecule. In the display technology, the EV-producing cells can be engineered with a plasmid in order to induce the expression of exogenous proteins. The post-loading method consists in the direct introduction of drug molecules into EVs after their isolation. (**4**) The engineered EVs may also be manipulated to be more bioactive and bioavailable and can be administered to patients for the MPM therapy.

Regarding EVs purified from foods, such as plant-derived or milk-derived EVs, despite that they can offer an easy and large-scale production and result particularly stable, may raise different concerns than human source-based therapeutics EVs. It has been shown that food-derived EVs are carriers of toxins and allergens that may determine immune reactions [166,167,168] (Figure 1).

Bioengineering applied to EVs is a tool used in order to induce EVs to uptake an exogenous cargo, and to drive it into specific body districts for therapeutic purposes (Table 1).

Actually, there are two main different approaches to manipulate EVs: a pre and a post-loading method. These two are technically and theoretically different. The pre-loading approach consists of using a pre-existing endogenous cargo as the therapeutic molecule [169] (Figure 1).

The pre-loading method is the easiest way to obtain therapeutic EVs, because the potential therapeutic molecule is already inside the vesicles. One of the main issues of this methodology is the impossibility to control the amount of cargo loaded into the EVs. If it is true that endogenous molecules, such as miRNA, have a high potential in therapeutic purposes, it is also true that for nucleic acids natural EV content is very poor [170,171,172] (Figure 1).

Therefore, it is clear that a deeper understanding of the sorting mechanisms that regulate the miRNA and other nucleic acids loaded into the EVs is very important: several research groups are trying to clarify this process, indicating for example Annexin A2 and Y-box binding protein 1 (also called as nuclease-sensitive element-binding protein 1) as two coworkers able to bind miRNA sequences mediating their EV’s loading (the first one in a sequence independent manner and the second one related with the binding with miRNA-223) [171,173,174].

These modifications made on EV-producing cells can be various and are somewhere between a “pre loading” and a “post loading” method. In fact, through genetic engineering techniques, it is possible to induce an overexpression of therapeutic molecules, such as miRNA, siRNA or proteins on the producing cell [171,175] (Figure 1). The fusion of therapeutic proteins with EV’s localized proteins is an efficient strategy that might drive the expression of the interest protein onto the EVs surface: this specific technique is called “Exosome display technology” [175,176]. Another promising approach is the use of specific functionalized domains in order to induce the expression of exogenous proteins: for this purpose the constant region one and two (C1C2 domain) of Milk Fat Globule-EGF Factor 8 Protein (also called Lactaderin) might be used, and promising works show the efficacy of this technique [171,176,177] (Figure 1).

The post-loading method consists of the encapsulation of therapeutic molecules on the EVs after their isolation. It is more efficient than the pre-loading one, because manipulating EV’s cargo after the isolation gives the operator a deeper control on the encapsulation efficiency (*EE*%) and loading capacity (*LC*%) of the final product. Those two are parameters used to calculate the efficiency of the final product in DDS [178,179], according to the following equations:

(1) $$\mathrm{EE}\%\;=\;\frac{Molecules\;correctly\;encapsulated\;with\;the\;drug}{Total\;amount\;of\;molecules}\times 100$$

(2) $$\mathrm{LC}\%\;=\;\frac{Molecules\;correctly\;encapsulated\;with\;the\;drug}{Amount\;of\;carrier\;that\;carries\;drug\;\left(EVs\;in\;this\;case\right)}\times 100$$

It is possible to intervene on EVs cargo through three different ways: chemical-treating EVs, through physical methods or using electroporation (Figure 1).

Sometimes the lipid bilayer prevents passive encapsulation of therapeutic compounds, so using chemical solvents in order to create pores on the lipid surface, or using some freezing–thawing cycles, can facilitate the drug loading into EVs [180,181]. In reality, the gold standard for the manipulation of EVs is electroporation: the electric pulse can induce the formation of pores on the surface of the vesicles, allowing migration of therapeutic compounds into them [177]. However, electroporation and approaches that affect EVs membrane stability and integrity present limitations due to the creation of aggregates, leading to an overestimation of the loading efficiency [135,182].

In cancer therapy, there are several examples of EVs encapsulated with chemotherapeutic drugs, such as doxorubicin or patitaxel, which are currently ongoing on preclinical trials [183,184].

It is important to underline how the choosing of bioengineering methods is strictly related with the properties of each compound used.

The possibility of exploitation of EVs as therapeutic nanocarriers has entered the field of anticancer therapy in the last decade, due to the similitude with liposomes, which have been studied for a long time for their capability to encapsulate drugs. Considering some physical features and the structure of the lipid envelope, EVs and liposomes appear similar [185]. Moreover, it is possible to synthesize liposomes so that they share many of the membrane properties of exosomes [186,187]. Despite this, although several liposome-based drug products are currently available in the market [188], they lack in selectivity and they can induce side effects, so much so that the clinical trials of liposomes turned out to be poorly effective [188]. Compared to liposomes, EVs have a unique lipid and protein composition, which favors targeting and organotropism. In fact, as mentioned above, EVs play a key role in cross-talking between cells and, due to the biogenesis mechanism, they have a very similar membrane to the cells that produced them; consequently, this would represent a characteristic to be exploited, for example in autologous therapy, to reduce renal clearance. In addition, immune compatibility, due to the EVs’ membrane features, constitutes an advantage that establishes them as a suitable target-therapy strategy, when compared with liposomes. Similarly, to liposomes, when injected intravenously EVs are rapidly cleared by the reticuloendothelial system in the liver and spleen, reducing drug efficacy. On the contrary, exosomes administered in a tumor in situ have a major rate of association to cancer cells and besides, autologous EVs show an enhanced organotropism [189]. In terms of immune compatibility between the EVs donor and receiving host, the allogeneic EVs applications seem to be feasible, for instance, taking into account the typical practices in hematopoietic transplantation (HLA, human leukocyte antigens-matching) [165,190,191,192]. Currently, strong evidence that can demonstrate the immune effects in allogeneic administration of EVs is lacking. Thus, the complete characterization of EVs and the source of EVs may lead to a robust definition of the mechanisms underlying the therapeutic effect of EVs [169].

The intrinsic ability of EVs to bind target cells, as mentioned before, is a particular point on targeted therapy. However, it is also possible to think to improve this natural feature, modifying the structure of those vesicles in order to improve the delivery to target tissues: for instance, it is possible to improve the blood circulation of EVs coating them with polyethylene glycol, or increasing the expression of CD47 on the vesicle surface, or using specific residues (like the C1C2 domain of Lactadherin) in order to conjugate specific antibodies on the EV surface, switching and driving the specificity of the complex [171,193,194].

An emerging drug delivery strategy, based on nanoparticles, that has demonstrated great potential in the treatment of different diseases and in regenerative medicine [135], is to create a hybrid carrier between exosomes and liposomes, in order to combine the features that may increase the effectiveness of anticancer therapies. These exciting new approaches consist of membrane fusion methods and have the aim to combine the high yield and high drug-loading capacity of liposomes with the EVs’ intrinsic properties, such as low immunogenicity, stability in circulation and high efficiency in target reaching and in tissue retention [195,196,197]. The freeze–thaw method has been used to induce the mixing of liposome and exosome membranes, which have been embedded with a specific membrane protein. This has determined the enhancing of the delivery function of the exosomes by changing the lipid composition or the properties of the exosome by membrane fusion [179,198]. This process seems to be exploiting the biocompatibility of EVs, allowing the camouflage of liposomes by enriching their lipid bilayer and inner compartment with biogenic molecules and on the other side, improve the EV drug loading, proper of liposomes [170,198]. More recently, the functionalization of exosomes, to increase their ability to deliver various contents into cells, has been successfully tested using nanogel systems that ensure their effective delivery in a functionally intact state [199]. Other interesting technologies have developed artificial nanovesicles, with features resembling those of EVs membrane and obtained from broken cells [200,201]. Despite the exciting potential of engineered EVs as drug delivery systems, the feasibility, and scalability of EVs and EVs-like systems are strongly dependent on some aspects that need to be addressed to accelerate the translation into the clinic. The issues that must be defined include the characteristic of the EVs source that entails the question of effectiveness and biocompatibility. Important are the isolation, storage and quality control procedures of EVs that affect the use of clinically relevant doses and EVs-based therapy safety. Moreover, ethical issues are noteworthy.

Considering the growing interest and the breakthrough data that are continuously provided in the field of EVs-based therapy, much progress is being taken to develop standardized protocols and to establish efficacy and biosecurity criteria, to the aim to translate this platform into reality to treat diseases like cancer.

## 4. Malignant Pleural Mesothelioma: The Lack of Efficient Therapeutic Strategies and Possible Drug Delivery Strategies

Malignant mesothelioma (MM) is an insidious neoplasm arising from mesothelial surfaces such as pleura and peritoneal cavities, pericardium and tunica vaginalis. The distribution of the tumor may involve lining cells in a continuous manner. Among all different subtypes of MM, MPM is the most common one [202,203,204,205,206].

MPM is an aggressive, rare tumor, with increasing incidence and poor prognosis. The massive use of asbestos after the Second World War exposed a significant number of people to asbestos, dramatically increasing the risk of developing MPM in 1960. Although the use of asbestos was significantly reduced or abandoned in the western world in the 1980s, because of the long latency between the exposure to the contaminant agent (asbestos) and the onset of the disease (from 15 to 60 years), mortality due to MPM continued to rise [207,208,209,210]. The global incidence of MPM is uncertain: this is due to a poor compatibility of databases from different countries, and few international data is available [208]. It is estimated that every year around 43,000 people die due to this tumor [202,208]. In Italy, according to epidemiological studies, MM represents 0.4% of the total amount of tumors diagnosed in men and 0.2% in women (AIRTUM). As mentioned before, in the last ten years there has been a significant increase in the diagnosis of this rare neoplasia, also due to the mean latency of the tumor of 44.6 years in 2544 cases diagnosed in the period from 1993 to 2001 [202,208].

A macroscopic observation is not enough for diagnosing MPM, as it is not always easy to distinguish between a benign pleural lesion and a malignant one. Certainly, the best way to evaluate the malignancy is to analyze the presence of tumor invasion. Furthermore, some MPM patients are not eligible for pleural biopsy, and diagnosis must be conducted on pleural effusions (PE) [211]. Considering the above, finding affordable biomarkers that can ease the recognition of malignant subtypes seems to be necessary. Unfortunately, current methods of detection, based on the most common alteration documented on MPM, lack in reliability and are generally not recommended. In fact, it is possible to say that currently there are no standardized diagnostic methods based on the most common genetic MPM’s alterations. Nevertheless, a transversal analysis conducted by Bruno R. et al. [212] has investigated the possibility to find new suitable biomarkers: first, the classical approach, involving standard immunohistochemical biomarkers such as glucose transporter 1, p53, desmin, epithelial membrane antigens and insulin-like growth factor mRNA binding proteins [211,212,213,214,215], has been combined with soluble PE biomarkers, then it has been evaluated through ELISA assay (mesotelin and fibulin-3). They have also evaluated new emerging biomarkers, recently introduced in the clinical practice (BAP1, breast cancer associated protein 1, through immunohistochemistry and p16 through, fluorescence in situ hybridization) [216]. Despite promising results, Bruno’s team has demonstrated that none of the tissues and soluble markers are highly sensitive enough to distinguish benign from malign pleural lesions. Only BAP1 and p16 showed a high specificity in discerning pleural lesions, both in PEs and tissues. Those two markers, BAP1 and p16, result exclusively unexpressed or deleted only in MPM. Considering that BAP1 and p16 are not deleted in all MPMs and their sensitivity can variate between 43% and 93% for p16 and 61–67% for BAP1, negative results can sometimes be inconsistent in order to exclude the malignancy [204,217,218]. Therefore, these markers may be not specific to MPM, but may aid diagnosis and may have prognostic significance [216,217,218].

Another important field of increasing interest concerns the link between MPM and EVs, especially exosomes. Currently, the role that exosomes, secreted by MPM, exert in tumoral progression, and their effector functions are increasingly being investigated. The analysis of samples of PE obtained from various patients proved to be enriched by EVs secreted by the tumor itself and, in particular, by exosomes [70,219,220]. However, currently, there are very few works in the literature about the function of exosomes secreted by the MPM. In order to identify the function and catalog the mesothelial-derived EVs, a close proteomic analysis was carried out on MM-derived exosomes. From an initial pool of 2178 proteins present in the exosomal isolate, all proteins common to all exosomes or proteins involved in their biogenesis (such as ESCRT or tetraspanins) have been excluded. Following this, it was possible to underline several proteins that constitute a sort of molecular “fingerprint” of MPM: the list of potential candidates as mesothelioma biomarkers includes, but is not limited to, Piruvate Kinase, Annexin A1 and A2, Heat Shock Cognate 71, Heat Shock Protein 90, Alpha Enolase, glucose6-phosphate1-dehydrogenase and 5 tubulin isotypes (such as TUBB4A, Q8IWP6 and B3KPS3) [219].

These and other potential candidates constitute the so-called “mesothelioma exosomal signature”, a potential pool of 570 proteins that can be used as markers for early and minimally invasive diagnoses, or as a basis for deepening the signaling mediated by the MPM-derived exosomes, and the way in which the secreted EVs influence the tumor microenvironment [219].

Anyway, as for the other tumor types, some specific functions mediated by the active secretion of exosomes by MPM have been analyzed and reported in the literature. In fact, it has been shown how MPM-derived-exosomes are involved in the formation of “tunneling nanotubes”, which are actin-based cellular extensions that act as channels for the transport of cellular material, in order to implement cell–cell communication [221]. In addition, MPM-derived exosomes also mediate immune evasion through the downregulation of the Natural Killer (NK) group 2 member D receptor, which is normally involved in the killing mediated by NK cells and by CD8+ T cells [222]. Finally, it has been observed how, through the exosome-mediated expression of CD39 and CD73, MPM can induce the extracellular expression of adenosine that, in turn, behaves as an immunomodulator exerting an anti-inflammatory action by suppressing the T-cell mediated response [223].

## 5. Future Perspective: The Challenge in the Exploitation of EVs in MPM Therapy

Concerning treatments, the handling and management of MPM therapies are a challenge for clinicians. First-line approaches include surgical intervention, chemotherapy or both of them. However, none of the actual standard therapies approved could be considered as an ultimate cure, but more as a palliative treatment. Moreover, currently, a standardized second-line therapy, meant as a different and effective treatment, does not exist and the overall survival rate after a two year follow-up remains poor [224,225].

In the oncological field, lines of treatment are dramatically important. These refer to different approaches to handle the cancer at different times. First line treatment is the first approach indicated by international guidelines. Other lines of treatment are usually exploited to treat tumors in which the first line approach does not work, side effects of the first line are not well-tolerated by the patient or new approaches seem to be more effective [226].

Therefore, the importance of new strategies that may lead to a more efficient and proficient handling of MPM is clear.

Several trials have been performed with the aim of increasing MPM treatment options for patients that respond negatively to the first line treatment.

An interesting ongoing clinical trial concerns the possibility of using pegylated phospholipid vesicles as a carrier for doxorubicin [227]. This study is proposed as a possible first-line treatment for the handling of MPM, by implementing the uptake of doxorubicin directly in the tumor site, consequently reducing the side effects of traditional systemic therapy, with acceptable toxicity values that improve the patient’s quality of life [227].

Another phase II study, conducted by Szlosarek et al., concerns the use of pegylated arginine deaminase (ADI-PEG20) [228]. In fact, preclinical studies demonstrate how arginosuccinate synthetase 1 (ASS1) enzyme deficient tumors are sensitive to arginine deprivation, leading to cell death. The trial showed that the progression free-survival (PFS) of patients treated with the compound ADI-PEG20 increased compared to the control group (15.7 months in the ADI-PEG20 group versus 12.1 months of PFS in the control group) [228].

Promising results also come from the use of a humanized anti-EGFR (epidermal growth factor receptor) antibody, Nimotuximab (h-R3) [61]. In the study in question, the researchers wanted to evaluate antibody biodistribution, uptake on the tumor site and binding affinity for EGFR. The study was conducted in vivo on MPM xenograft models, radiolabeling the antibody with Gallium-67, demonstrating a remarkable uptake of the antibody-radiolabeled complex at the tumor site [61].

Concerning the above-mentioned possible therapeutic tools in drug delivery, it might be tempting to consider EVs, and exosomes more specifically, as a potential therapeutic tool in the handling of MPM. Nevertheless, the small amount of works in the literature leads us to further investigate the connection between EVs and MPM. In this regard, once the effective relevance of the production of exosomes by the MPM has been assessed, it would be interesting to discover whether it is possible, and if so how, to exploit this system of cancer communication in order to deliver exogenous molecules, possibly with therapeutic functions, using the natural tumor network to attack itself with its own messengers.

The idea we want to propose is the exploitation of EVs isolated from primary cell cultures of MPM, in order to be able to modify them through post-loading techniques. In this way, even though the isolation efficiency might be lower if compared to therapies with recombinant proteins (>5 g/L of culture medium) [171,229], the final product would be far more representative of the actual secretome of the MPM. This would allow an expression of surface receptors of tumoral exosomes closer to the physiological one; it would also theoretically allow a more efficient drug loading, through post-loading techniques, and drug delivery.

Among other things, exosome surface proteins define their tropism, and today the pivotal role of EVs on tumor signaling is largely confirmed [230].

Due to the relative simplicity and low cost of EV manipulation techniques, they represent a potentially suitable model of DDS. The prospect of attacking the tumor by exploiting its own messengers is not only fascinating from a scientific point of view, but also clinically remarkable: the biocompatibility of a biopharmaceutical like this, based on a loading into the vesicles of a drug, it would be complete.

In addition, unlike the strategies adopted in other drug delivery models, in which specificity is entrusted to a single molecule present on the surface of the target cell (e.g., monoclonal antibodies and peptide-based nanocarriers), EVs can rely on a much more layered and complex vehiculation system. Of course, a careful evaluation of the tumor microenvironment, the choice of an ad hoc delivery system and the electric charge of the nanocarrier have to be taken into account.

## Figures and Tables

**Table 1:** The advantages and disadvantages of main drug delivery approaches. Abbreviations: ADC, antibody-drug conjugated; EVs, extracellular vesicles.

Strategy	Advantages	Disadvantages	References
**Nanoparticles**	• Flexibility • High stability in vivo • Increased compound half-life • Hydrophilic and lipophilic compounds can be both loaded • Enhanced permeability of tumor vasculature facilitates nanoparticle delivery on the tumor site	• Depends on the nanoparticle type • Inorganic nanoparticles may trigger the immune-system	[19,20,21,22,23,24,25,26,27,28]
**Liposomes**	• Similarity with cell membrane • High variety of drug encapsulation • Low systemic toxicity	• Sensitivity to sterilization methods • Low stability in circulation • Low reproducibility in liposome loading and size control • Short shelf-life	[29,30,31,32,33,34,35,36,37,38,39,40,41,42,43]
**Polymer Conjugated Drugs**	• Increased compound half-life • Increased high-dose drug tolerance • Increased specificity	• Deep knowledge of polymer–receptor molecular interactions required • Poor information about long-term side effects	[44,45,46,47,48,49,50,51,52,53,54]
**Small molecules, peptides and antibodies**	• Various anticancer effects • High cell permeability • Low systemic toxicity	• Depends on the small molecule type • Size-influenced pharmacokinetics • Short half-life	[55,56,57,58,59,60,61,62,63,64,65,66,67,68,69]
**ADC**	• High specificity • Remarkable results for AB working as single entities	• May trigger the immune system • High production costs • Some clinical trials showed no significant improvement of patient outcomes compared to canonical therapies	[4,70,71,72,73,74,75,76,77,78]
**Reconfigurable Organisms**	• Extremely configurable organisms • High drug loading efficiency • Autonomous, self-repairing system	• Machine learning is still unripe • Research in this field is still in the embryonic stage	[79]
**EVs**	• High biocompatibility due to the endogenous origin • High specificity • Can be highly modified in order to enhance or modify tissue specificity • Due to the phospholipid bilayer, they can easily fuse directly to the target’s plasma membrane • Presence of specific surface markers that preserve them from phagocytosis • Functional complexity, not easy to artificially replicate	• Generally low efficiency of EVs isolation methods • Lack of clinical evaluations • Lack of standardized isolation methods • Deepening of the understanding of intracellular production/packaging mechanisms still ongoing	[80,81,82,83,84,85,86,87,88,89,90,91,92,93,94,95,96,97,98,99,100,101,102,103,104,105,106,107,108,109,110,111,112,113,114,115,116,117,118,119,120,121,122,123,124,125,126,127,128,129,130,131,132,133,134,135,136,137,138,139,140,141,142,143,144,145,146,147,148,149,150,151,152]
